# Prognostic Significance of Solitary Lymphnode Metastasis and Micrometastasis in Gastric Cancer

**DOI:** 10.3389/fsurg.2018.00063

**Published:** 2018-10-18

**Authors:** Konstantinos D. Mpallas, Vasileios I. Lagopoulos, Apostolos G. Kamparoudis

**Affiliations:** 5^th^ Surgical Department, Aristotle's University of Thessaloniki, Hippokrateio General Hospital, Thessaloniki, Greece

**Keywords:** gastric cancer, solitary lymphnode metastasis (SLN), micrometastasis, skip metastasis, prognostic factors

## Abstract

Gastric cancer (GC) used to be one of the most common malignancies in the world and still is the second leading cause of malignancy-related death in the Far East. The most significant factors that were found to be associated with the clinical outcome in patients with non-metastatic (M_0_) gastric cancer is tumor's depth of invasion, the presence and the extend of lymphnode involvement, as well as the histological type according to Lauren (intestinal or diffuse). Although it is generally accepted that D2 gastrectomy is the procedure of choice to achieve adequate oncologic excision, there are quite many concerns for its use in patients with early gastric cancer (EGC), where N_o_ or N_1_ specimens are frequently reported. The last two decades, with the evolvement of cancer cell detection techniques, the attend of the medical community is focused on GC patients with solitary lymphnode metastasis (SLN) or micrometastasis (mM). There is a discussion whether SLN should be attributed as the “real” sentinel node (SN) and its projection on patients' survival. The aim of this study is to review the recent literature and attempt to clarify the clinical significance of SLN in gastric cancer.

## Introduction

Surgery is the cornerstone of treatment in patients with gastric cancer (GC). The therapeutic effect of the surgical approach was found to be proportional to the extent of excision of epi- and perigastric lymphatic tissue, leading to operations that resect en bloc the lymph nodes that drain the stomach, decreasing that way the possibility of recurrence. The Japanese colleagues have been pioneers in the study of gastric cancer, mapped and organized in basins the lymph node stations surrounding the stomach, and standardized the gastrectomy procedures according to the wideness of lymphatic resection to D1, D2, and D3. The arrival of less invasive treatments for early GC (T_1a_ T_1b_), such as endoscopic mucosal resection (EMR) or endoscopic submucosal dissection (ESD) raise the question of lymph node status (1). Many studies have shown that, even in early GC, there is high possibility of cancerous migration to the lymph nodes and it's not uncommon that this migration occurred to just one node, the so called solitary lymphnode metastasis (SLN). In some cases, the cancer spread in the lymph node does not exceed 2 mm, and thus called micrometastasis (mM) (2), while in other node specimens the malignant portion is fewer than 0.2 mm in diameter, considered to be isolated tumor calls (ITC's).

## Materials and methods

To identify related studies and published literature the authors performed independent search in the electronic databases MEDLINE, Embase and PubMed. The following Medical Subject Headings (MeSH) were used in the search: “solitary lymphnode metastasis,” “gastric cancer,” “micrometastasis,” “lymphatic drainage,” and “prognostic factors.” Solely articles published in English from 1998 until 2018 were included. Next, by screening the title and abstract the related literature was further selected, and the articles chosen were thoroughly analyzed. Additionally, some references from these articles were also retrieved and added, to become more comprehensive.

## Detection of lymph node metastasis in GC

### Preoperative evaluation

Every gastric cancer patient nowadays is examined thoroughly for metastatic disease. In order to set the correct therapeutic scheme, it would be more than useful to know preoperatively the lymphatic spread of the disease. Currently, the evaluation of lymphnode involvement is mainly performed by multislice spiral CT scan (MSCT) and endoscopic ultrasound (E-US). Recent reports place the diagnostic accuracy of these methods for LN status in GC around 61% for MSCT and 76% for E-US (3). It is obvious that these results are not satisfying, but are the best we have. In 2015, a report from Ma et al was published (3), proposing an equation that predicts lymphnode involvement using preoperative parameters (CEA levels, Tumor size, T staging by CT, LN status by CT). The authors support that this equation is capable of allocating correctly 85% of the GC patients according to LN metastasis or not (91% in SLM subgroup), but this finding hasn't been confirmed by other researchers yet.

### Intraoperative assessment

In everyday clinical practice the majority of surgeons perform a D1+ gastrectomy in order to treat GC. Although usually all the preoperative assessment regarding the extend of the disease is performed, it is not uncommon that intraoperative findings require an immediate histological examination. That is most likely to be asked for a suspicious lymphnode, especially outside the planned excision margins, and the pathologist has 5–10 min to examine random sections of the node under traditional heamatoxylin-eosin (HE) staining. Inevitably, in the post-op pathology report where proper serial sectioning of the node is performed, there is a respectful number of patients that show metastasis in lymphnodes thought to be negative for cancer intraoperatively (false negative). Furthermore, there are similar studies in patients with breast cancer that demonstrated up to 43% (4) false negative results regarding the intraoperative examination of LN status.

**Figure 1:**
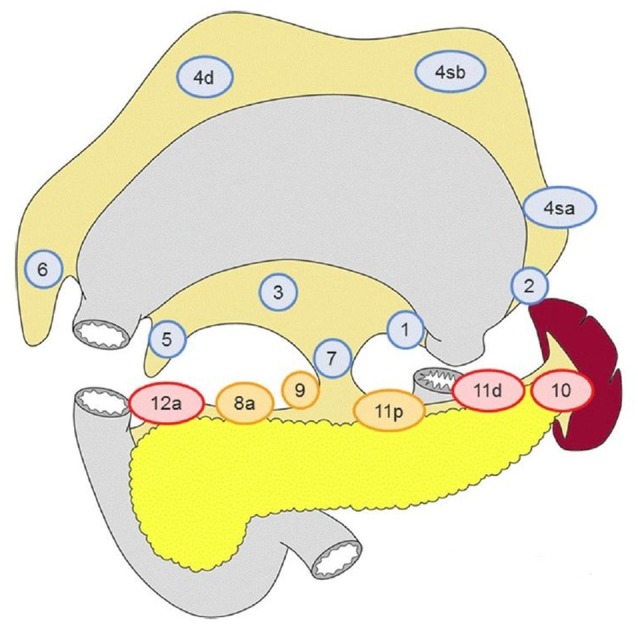
Lymph node stations of the stomach. Stations 1–7 are adjacent to the gastric wall and are referred as level I, while stations 8–12 are referred as level II. Adopted from Japanese Gastric Cancer Association (8).

For that reason, the concept of sentinel node that has been used successfully in breast cancer and melanoma, was proposed to be applied in gastric cancer. Indeed, there were studies that the sentinel node navigation in GC was tested intraoperatively (either with endoscopic infusion of dye around the tumor and/or with use of radioisotope/indocyanine green) (5, 6) but the results were controversial and surely not comparable to the ones from the breast cancer and melanoma reports. Perhaps the most interesting findings of these studies is that the distribution of lymph node metastasis usually occurs to the adjacent lymphatic basin (according to Japanese Gastric Cancer Association) rather than a single -sentinel- node and that there are cases that the cancerous spread may affect distant perigastric node stations (level 2) (7), while local lymphnodes (level 1) (8) are found negative, leading to skip (or jumping) metastasis (SM) (Figure 1). A subtype of skip metastasis are *transverse metastases*, where a tumor located in the lesser curvature is accompanied with metastasis on lymph nodes of the greater curvature and vice versa.

### Newer molecular methods to detect cancer cells

Immunohistochemistry (IHC) was a major breakthrough in the pathology field that changed the way fellow pathologists handle the surgical specimens. In gastric cancer, IHC finds its use mainly with antibodies against cytokeratin (AE1/AE3) and less frequently with antibodies against epithelial membrane antigen (EMA) for the detection of LN metastasis. The advantage of this technique is that it recognizes small clusters of few cancer cells even ITC's, that would slip through under usual HE examination (9); the majority of mM as well as ITC's are detected that way. The main disadvantage of IHC is that is a time-consuming technique so that it cannot be easily applied intraoperatively. Although Matsumoto et al. (10) developed a fast IHC assay that can identify LN metastasis in just 30 min, still it has not been tested in wide use. Isolated tumor cells ITC's are almost exclusively detected by IHC or rt-PCR. In the current staging system ITC's are not characterized as pN1, but as pN0 mol(+).

The latest addition in our investigating tools is reverse-transcription Polymerase Chain Reaction (rt-PCR). By this technique we examine lymphnodes in real time for target markers, such as CK, CEA, or MUC2 protein, that imply cancerous invasion. There are quite many studies where patients that were node negative both with HE and anti-CK immunoassay, cancerous load was finally visible when rt-PCR was used (11). The sensitivity of rt-PCR is outstanding (>90%) (12), but is also demanding in time so it cannot be used in everyday clinical practice, plus it has false positive results. Undoubtedly, it is a promising method for the future, where minimal invasive surgery combined with individualized lymphadenectomy will probably prevail. Indeed, the concept of endoscopic resection of early GC with targeted surgical resection of lymphnodes seems to gain place (13).

## Incidence of solitary lymphnode metastasis (SLM)

Most of the data we have on the incidence of SLM in gastric cancer come from studies that examined retrospectively surgical specimens of patients characterized as TxN0 under routine pathological examination (HE staining), that were reevaluated with IHC. Most researchers report rates of SLM ranging from 10 to 42% (5, 7, 11, 14, 15), with the lower values found usually in early GC patients. Indeed, there is evidence that the possibility of SLM raises as the tumor invades deeper in the gastric wall, infiltrating the submucosal lymphatic tissue. Interestingly, it was found that the SLM was not the larger lymphnode of the station (60% < 5 mm) and the intranodal cancerous foci were mostly arranged the marginal-sinus type (16). That finding is constant with the data from the recent meta-analysis of Zhao et al. (17) concerning early gastric cancer treated by endoscopic resection and additional surgery: around 10% of the patients with T1 tumors had malignant infiltration of lymphnodes, and the possibility of lymphatic involvement raises with the vertical spread of the tumor and lymphovascular invasion of the submucosa.

In the 35-year retrospective study by Tokunaga et al. the anatomic location of SLM was studied in patients that underwent D1+ gastrectomy for serosa negative (<T3) GC. They reported that in 90% of the cases SLM is detected adjacent the left gastric artery (stations 1, 2, 3, and 7) or along the right gastroepiploic artery (stations 4d, 6, 14v), stating though that the distribution depends on the location of the primary tumor (6).

Over half of SLM cases, reflect micrometastasis (mM) that are detected with newer methods, basically with IHC and occasionally with rt-PCR (where available). Fukugawa et al. conducted a retrospective study on GC patients treated with partial or total gastrectomy and systematic lymphadenectomy (D1+ or D2) and reexamined the lymphnodes with IHC (18). Interestingly, the application of IHC in 107 pT_2_-T_3_ node-negative patients revealed that 38 (35.5%) of them had cancerous load in at least one lymphnode. On the same path, Yano et al. (5) in a well-structured study with standardized lymphadenectomy (D1 for cT1, D2 for cT2) focusing on sentinel node navigation surgery found out that half of the patients with mM, as diagnosed with IHC, where misdiagnosed as N0 with HE. There are older reports that raise the incidence of micrometastasis detected by immunohistological assay up to 43.2% of patients with T2 GC (14). Moreover, Kubota et al. (19) tested the detection rates of rt-PCR and IHC in 304 GC lymphnodes from 21 randomly chosen GC patients (T1-T4) that were mostly treated with extended (D2-D3) lymphadenectomy. He reported positivity rates of 9.9% with rt-PCR and 3.6% with IHC in nodes negative under conventional histology. Sonoda et al. (20) examined 305 lymphnodes of 28 GC patients that were offered curative surgery with haematoxylin-eosin, IHC and rt-PCR. Again, 49 nodes that were found negative under microscopical analysis, were found to carry metastasis with rt-PCR for MUC2 protein. From these 49 nodes, only 6 were detected by IHC, while no node was tested positive with IHC and found negative on PCR. So, it is quite logical to assume that wider appliance of rt-PCR will reveal a higher incidence of mM and consequently SLM, as in the past studies many nodes carrying mM were probably eluded.

### Incidence of skip metastasis

A significant portion of SLM (reports range 10 to 60%) (3, 5, 6, 9, 12, 21) is not detected on level 1 nodes, but in level 2 nodes leading to so-called skip metastasis. SM usually occur along the left gastric artery, anterior common hepatic artery but can also be cited along coeliac or splenic artery. Huang et al. (22) reported that transverse lymphnode metastasis were found in as high as 50% of patients with tumor located on the greater curvature, but the inclusion criteria on this study, as well as the surgery performed, have some blurred spots. There are many theories regarding the explanation of this phenomenon, based on the lymphatic drain of the stomach (23, 24), the possible “hostile” microenvironment of level 1 nodes (24), obstruction of the main lymphatic rout by cancer, low concentration of certain adhesion molecules, etc. From the review of the literature we assume that the real percentage of true skip metastasis must be significantly lower (between 5 and 15%) for the following reasons: (i) the majority of studies on skip metastasis were retrospective and only in few of them rt-PCR or IHC was used, thus level 1 node involvement (mM) was probably present but not identified. This is constant with the finding by Arai et al. (16), where in 7 cases thought to have SM, the careful re-examination and immunohistochemistry showed that in 3 out of 7 patients level I nodes was also invaded. (ii) there are research articles (25) that suggest the existence of lymphatic routes leading directly from the gastric submucosa to level II nodes, especially in the posterior wall, so in this case SM rather represent the sentinel node than reflect true jumping metastasis. To support this further, in the recent work of Aoyama et al. (26) regarding sentinel node navigation surgery on early GC they found invaded lymphnodes outside the local lymphatic basin in just 8 out of 359 patients.

## The effect of SLM on the prognosis of gastric cancer patients

### Current state

The significance of SLM and its effect on the prognosis of the GC patient is still a matter of debate. Many recent studies were focused the field of SLM, especially in the early GC patients under the sentinel node concept, but end up to controversial results. Li et al. (14) discovered statistical significance in 5 years survival (80.5 vs. 90.2%) between SLM (+) and SLM (–) in 145 gastric cancer patients that the specimen contained more than 15 lymphnodes and found correlation of SLM presence with tumor's depth of invasion. The number of lymphnodes retrieved gives an idea on the extend of lymphadenectomy, but not for the completeness of it, as Karavokyros and Michalinos demonstrated on a solid review on D2 lymphatic excision (27). Another limitation of Li's study was that lymphnodes were examined solely by histology and heamatoxylin-eosin, therefore it's logical to assume that mM was not identified.

The 5-year overall survival rates were significantly worse for pT2-T3N0 patients with mM in the pioneer well-structured study of Yasuda (66 vs. 95%) (28), while presence of mM was independently associated with worst prognosis in pT1N0 patients in the work of Cao et al. (29). The latter study though, has a lower credibility than that of Yasuda, as it states that patients enrolled had early GC and they were treated by curative (R0) gastric resection with lymphadenectomy without any further information. Anyway, their reports on 21.3% incidence of lymph node mM in patients with node negative early gastric cancer cannot be ignored.

Solitary macro and micro metastatic lymph node involvement were significant risk factors -both in univariate and multivariate analysis- for recurrence in the study of Lee et al. (9). In their work regarding GC patients treated with surgery + extended lymphadenectomy (average LN retrieved ≈ 40), report that 36% of the nodes negative under HE were found to have mM on IHC. By comparing the survival curves between mM (–) vs. mM (–), they propose that mM should be regarded as N+, as this modification “corrects” the intersection of the survival curves of N2-N3a patients of their study. Therefore, some patients will have to be considered for stage migration. Additionally, Yanagita in his study on sentinel node in GC patients (30) discovered that even mM has high proliferative potential, so probably there is no real difference in the clinical outcome whether we deal with macro or micro metastasis at a single lymphnode.

On the contrary, a number of researchers support the SLM has little or no effect in patient outcome. In the single-center study of Morgagni et al. (31) 300 patients with EGC (pT1N0) were treated with D2 gastrectomy over 25 years. The average number of nodes removed was 18—less than expected for a D2 lymphatic removal, some of them with evident mM in the histology. The patients were followed up for 5 and 10 years with no statistical significance in survival, so they concluded that—at least for EGC-solitary micrometastasis does not affect prognosis. No difference in 5-year cancer-related deaths were reported also by Kim et al. (32): In this well-designed study, among 90 patients with EGC (pT1a-T1bN0) 10% had solitary mM, there were 10 deaths during follow up and no recurrence. Similar findings were also shown for pT2-T3N0 patients with mM from Fukugawa et al. (18): 94 vs. 89% 5-year survival and 79 vs. 74% 10-year survival with no statistical significance. It should be noted though, that nearly all patients from these reports have been submitted to wide lymphatic resection (at least D2 gastrectomy), minimizing that way the possibility of lymphatic residual disease (33).

### Further considerations

In order to investigate the impact of solitary mM on the clinical outcome of patients with GC, Zeng, Zang and Dai conducted a meta-analysis (34) containing data from 12 cohort studies. The results were mixed, as the studies included had significant heterogenicity regarding lymphatic removal and cancerous invasion detecting methods. They did not find statistical significance for 5-year survival, but there were differences on recurrence rate, with a relative risk of 7.3 for the SLM+ group.

The projection of skip metastasis on survival of GC N1 patients is a confusing topic that needs to be clarified. The investigation of the multidirectional lymphatic flow of the stomach revealed pathways that connect certain submucosal regions directly to distal level II nodes (7, 24, 35) mainly along LGA or RGEA and almost always in the same lymphatic basin (6). So, true skip metastasis must be quite rare, less than 10%, and usually included in a standard D2 gastrectomy (35). Secondly, as shown by Li et al. (14) it does not have an impact on 5 years survival whether the SLM is a skip one or not. So, one could suppose that it is the positivity of the node that makes -if any- impact on the prognosis and not the location. That hypothesis is challenged though by the newer findings of Takeuchi et al. (36) where presence of invaded lymphnodes outside the sentinel lymphatic basin was associated with worst outcomes.

Stage migration is perhaps the most significant factor that influences survival in GC patients with SLM (37, 38). The presence of an invaded lymphnode -which probably must be attributed in mM too (38)—classifies the patient as N1, so that a T1 patient has a IIa disease instead of stage I the same applies for a T3 patient, identification of one lymphnode with cancerous foci over 2 mm should be characterized as having a stage III disease.

Amongst the studies included in our review, there were significant differences in the number of lymph nodes retrieved, therefore the presence of SLM probably had variant significance. Some authors preferred to use the SLM as percentage of the nodes harvested, referred as “MLR-metastatic lymphnode ratio,” and found out a threshold associated with negative prognosis when MLR is over 0.06 (39). An interpretation could be that if a SLM is found and the total number of nodes retrieved exceeds 16, it is most likely that it will not affect prognosis; but this finding has yet to be confirmed in a more powerful clinical study.

## Conclusions

Presence of metastatic foci on a single lymph node in gastric cancer patients is not uncommon, but not as frequent as it was thought to be, as shown by the advent of the newer detection methods. The identification of solitary lymphnode metastasis in GC seems to be correlated with tumor's depth of invasion, diffuse type according to Lauren (5, 40) and is associated with increased risk for recurrence and possibly with worst survival rates. The level of infiltration of the gastric wall is an independent negative prognostic factor by itself, so it must be clarified whether the unfavorable outcomes are due to the tumor's bad characteristics or due to the presence of SLM. Anyway, we think that existing literature favors the concept that SLM is associated with higher recurrence rates in GC patients, and that must be taken in mind when treatment plan is decided.

Based on the current data, we may assume that identification of SLM probably affects the prognosis of gastric cancer patients in case they have been treated with inadequate lymphatic dissection or if the tumor's biology shows aggressive behavior. In case of a standard D2-D2+ gastrectomy, the detection of SLM probably does not affect survival, as more likely the district lymphatic basin is included in the specimen (27, 41). In patients with EGC there are worries that this approach might be considered overtreatment, exposing the patients to possibly unnecessary higher morbidity and mortality rates (33, 42). The worry voices have a point that D3 gastrectomy has failed to prove significant superiority over D2 regarding survival rates (43). Still, in order to abandon a successful approach, newer techniques have to prove their safety and show -at least- equal outcomes.

The treatment of the patient suffering from gastric cancer is evolving to minimal approaches (44). The question of oncological adequacy raises every time lesser excisions are proposed, especially regarding the completeness of the lymphatic removal. So, it is crucial to define further the importance of solitary lymphnode metastasis with well-structured studies that will use standardized surgical approach, especially regarding the extend of lymphadenectomy. Sentinel node navigation surgery with intraoperative molecular diagnosis of lymphnode involvement is perhaps the most useful tool we currently have, in order to clarify this puzzling topic.

## Author contributions

KM and AK did the literature research, selected most of the references and addressed the line-up of the article. VL has written the manuscript and performed additional research, while the final review was executed by KM and VL.

### Conflict of interest statement

The authors declare that the research was conducted in the absence of any commercial or financial relationships that could be construed as a potential conflict of interest.
